# Transcriptome and microbiome of coconut rhinoceros beetle (*Oryctes rhinoceros)* larvae

**DOI:** 10.1186/s12864-019-6352-3

**Published:** 2019-12-09

**Authors:** Matan Shelomi, Shih-Shun Lin, Li-Yu Liu

**Affiliations:** 1 Department of Entomology, National Taiwan University, No 27 Lane 113 Sec 4 Roosevelt Rd, Taipei, 10617 Taiwan; 2 Institute of Biotechnology, National Taiwan University, Taipei, Taiwan; 3 Department of Agronomy, National Taiwan University, Taipei, Taiwan

**Keywords:** Transcriptome, *Oryctes*, Rhinoceros beetle, Cellulase, Microbiome, Antimicrobial peptides

## Abstract

**Background:**

The coconut rhinoceros beetle, *Oryctes rhinoceros*, is a major pest of palm crops in tropical Asia and the Pacific Islands. Little molecular data exists for this pest, impeding our ability to develop effective countermeasures and deal with the species’ growing resistance to viral biocontrols. We present the first molecular biology analyses of this species, including a metagenomic assay to understand the microbiome of different sections of its digestive tract, and a transcriptomics assay to complement the microbiome data and to shed light on genes of interest like plant cell wall degrading enzymes and immunity and xenobiotic resistance genes.

**Results:**

The gut microbiota of *Oryctes rhinoceros* larvae is quite similar to that of the termite gut, as both species feed on decaying wood. We found the first evidence for endogenous beta-1,4-endoglucanase in the beetle, plus evidence for microbial cellobiase, suggesting the beetle can degrade cellulose together with its gut microfauna. A number of antimicrobial peptides are expressed, particularly by the fat body but also by the midgut and hindgut.

**Conclusions:**

This transcriptome provides a wealth of data about the species’ defense against chemical and biological threats, has uncovered several potentially new species of microbial symbionts, and significantly expands our knowledge about this pest.

## Background

**Fig. 1:**
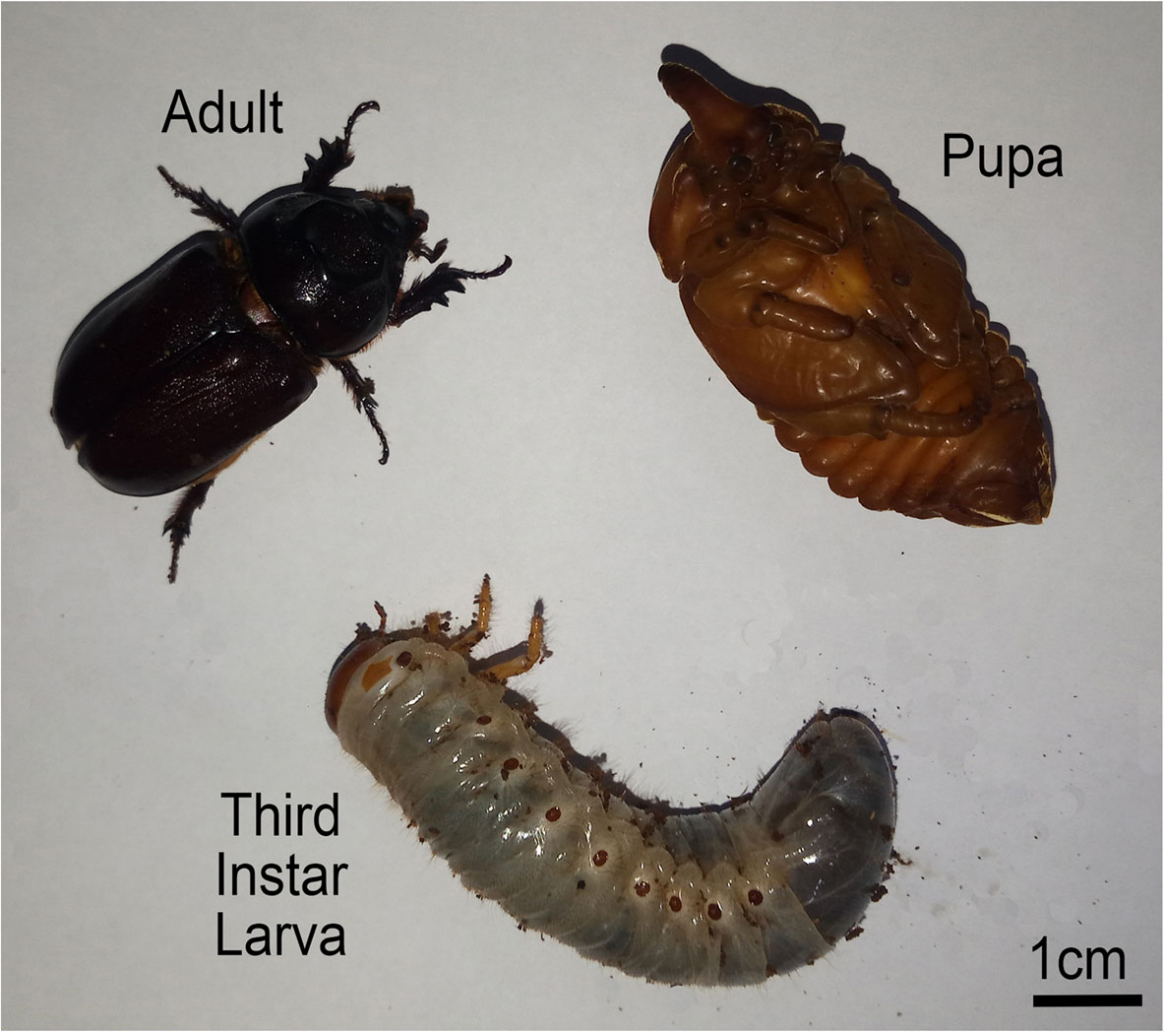
The Coconut Rhinoceros Beetle, *Oryctes rhinoceros.* Adult, pupa, and third (final) instar larva are shown. Scale bar is 1 cm. Photo credit: M. Shelomi

The Asiatic or coconut rhinoceros beetle (*Oryctes rhinoceros* L.) (Fig. 1) is a pest of palm trees in tropical Asia and the Pacific Islands. It is one of the most damaging pests of coconut and oil palm in these regions, and also attacks date, sago, betel, and raffia palms as well as banana, sugar apple, pandanus, and several ornamentals [1]. It is listed on the Global Invasive Species Database and has travelled as far east as Hawai’i [2]. The adults mate and the females lay eggs in rotten stumps or standing palms where the larvae develop. The adults are the most damaging stage, cutting into the palm crown and uncurled fronds to feed on plant juices [3].

The pest is mainly controlled through mechanical removal of adults. Fungi (*Metarhizium anisopliae* M.) can kill the pest under certain conditions, as can nematodes and the *Oryctes* baculovirus [4], however a virus-immune haplotype of the beetle has been described [5], reducing viral effectiveness overall [6]. Part of the beetle’s immunity includes antimicrobial peptides (AMPs), such as defensin [7], scarabaecin [8], oryctin [9], and rhinocerin [10]. Studying these peptides not only helps us understand the beetle’s defenses against potential biocontrol pathogens [11], but also may have applications in medicine through the constant search for new antimicrobials [12].

Another potential application of the beetles’ molecular biology is for plant cell wall degrading enzymes (PCWDEs) such as cellulases and hemicellulases [13]. These enzymes have great potential for biofuel production, and scarab digestive tracts have already been highlighted as potential sources of enzymes for bioreactors [14]. These, plus any immune system, xenobiotic metabolism, or detoxification enzymes [15, 16], would also be targets for next generation insecticides such as RNAi [17]. Disabling the larval ability to detoxify plant secondary compounds or chemical insecticides [18] or their ability to digest food could prove fatal. The possibility exists that *Oryctes rhinoceros* depends on symbiotic microbes for digestion, especially the production of PCWDEs [13, 19, 20]. Any symbionts would also be targets for control, as knocking out an obligate symbiont with antimicrobials is an effective control of the host insect [18], plus symbionts themselves can be used to mediate RNAi delivery for bioncontrol [21].

Molecular data on *Oryctes rhinoceros* is sorely lacking, with the closest being the draft genome of *Oryctes borbonicus* [22]. A nuclear and mitochondrial DNA population genetics analysis across its range from Thailand to Hawai’i found minimum variation, concurrent with rapid invasion but also suggesting that the genetic data from beetles in one part of the Pacific will be the same for as those from beetles across its range [2]. With the goal of understanding the basic biology of *Oryctes rhinoceros*, focusing on their potential symbioses as well as their digestive, detoxification, and antimicrobial genes, we ran the first next-generation sequencing study of the species. We here present the first metagenomic data on the microbial community of *Oryctes rhinoceros* larvae, and a transcriptome for the gut and fat bodies, which are the primary tissues involved in insect digestion, detoxification, and immunity. This data increases our knowledge of how *Oryctes rhinoceros* works on a molecular level, and identifies new targets for control of this invasive pest.

## Results

### Microbiome

Microscopy revealed that the hindgut and midgut contents were both rich in microbes. Two species were successfully cultured from the wood pulp in which the larvae grew. One (Orhi1, GenBank Accession Number MN089572) formed round, white colonies with irregular edges and a matte, rough surface and was identified as *Bacillus cereus* (Firmicutes: Bacillales) (100% 16S rDNA sequence similarity to *Bacillus cereus* ATCC 14579, GenBank Accession Number NR_074540.1). The other (Orhi2, GenBank Accession Numbers MN089573–4) formed round, off-white colored colonies with smooth edges and a glossy surface, and was identified as *Citrobacter koseri* (Gammaproteobacteria: Enterobacteriales) (> 98.9% 16S rDNA sequence similarity to *Citrobacter koseri* strain CDC-8132-86, GenBank Accession Number NR_104890.1).

The results of the metagenomic microbiome analysis are as follows. After removing one ambiguously identified OTU (“Bacteria”), a total of 43 OTUs were identified by QIIME2 from the beetle guts and/or wood pulp, with the majority identified as uncultured microbes (Table 1). Few sequences could be identified to genus with QIIME2, so all OTU sequences (trimmed to 400 bp sequences) were re-analyzed with BLASTn. A few still could not be identified to genus, with 16S sequences < 90% similar to any in the NCBI 16S rDNA database and likely representing genera new to science. One OTU identified only as *Bacillus* sp. from the metagenomics assay is 99.78% identical to Orhi1, and so is likely the same *Bacillus cereus*. One OTU identified only as “Enterobactereacea” by QIIME2 was identified as *Citrobacter koseri* by BLASTn and is 99.75% identical to Orhi2, and so is likely the same *Citrobacter koseri.* The latter was also found in the negative control, however.

**Table 1:** Microbial Taxa in the *Oryctes rhinoceros* Fat Body, Gut contents, and Surroundings

Phylum; Order	Closest Identifable taxon, % identity	Fat Body	Hind-gut	Mid-gut	Wood Pulp	diH_2_O
Euryarchaeota; Methanobacteria	*Methanobacterium beijingense* 97.72%	0	22	0	0	0
Euryarchaeota; Thermoplasmata	Methanomethylophilaceae > 80%	0	31	0	0	0
Actinobacteria; Actinobacteria	*Tsukamurella serpentis* 98.23%	0	12	0	0	0
Actinobacteria; Actinobacteria	*Cellulomonas fimi* (and others) 98.49%	0	0	0	96	0
Actinobacteria; Actinobacteria	*Gryllotalpicola soli/kribbensis* 98.99%	0	0	0	26	0
Bacteroidetes; Bacteroidia	Bacteroidetes > 80%	10	0	0	0	0
Bacteroidetes; Ignavibacteria	Melioribacteraceae > 80%	0	10	0	0	0
Chloroflexi; Anaerolineae	Anaeolineaceae > 90%	0	14	0	0	0
Chloroflexi; Anaerolineae	Anaeolineaceae > 90%	0	23	0	0	0
Elusimicrobia; Endomicrobia	*Endomicrobium* > 95%	0	10	0	0	0
Firmicutes; Bacilli	*Bacillus cereus* 99%	261	48	21	18	0
Firmicutes; Bacilli	*Bacillus drentensis* 99%	0	10	0	0	0
Firmicutes; Bacilli	*Lysinibacillus sphaericus* 99%	14	262	28	0	0
Firmicutes; Bacilli	*Trichococcus alkaliphilius* 99.50%	0	0	0	10	0
Firmicutes; Bacilli	*Enterococcus termitis* 98.73%	0	0	93	0	0
Firmicutes; Bacilli	*Lactobacillus sakei* 99.50%	53	0	0	10	0
Firmicutes; Bacilli	*Lactococcus taiwanensis* 95%	2751	86	27	42	0
Firmicutes; Clostridia	*Clostridium saccharoperbutylacetonicum* 99.24%	50	0	0	0	0
Firmicutes; Clostridia	*Clostridium sporogenes* 96.2%	16	0	0	0	0
Firmicutes; Clostridia	*Clostridium intestinale* 97.63%	65	52	0	151	0
Firmicutes; Clostridia	*Clostridium homopropionicum* 97.22%	61	0	0	0	0
Firmicutes; Clostridia	*Intestinimonas butyriciproducens* 96.70%	10	91	0	0	0
Firmicutes; Clostridia	*Oscillibacter* 93.67%	18	0	0	0	0
Firmicutes; Clostridia	Ruminococcaceae > 90%	0	13	0	0	0
Firmicutes; Clostridia	Ruminococcaceae > 90%	0	30	0	0	0
Firmicutes; Negativicutes	Ruminococcaceae > 90%	30	14	23	0	0
Fusobacteria; Fusobacteriia	Leptotrichiazeae > 80%	0	27	0	0	0
Gemmatimonadetes; Gemmatimonadetes	Gemmatimonadaceae > 80%	0	0	0	14	0
Patescibacteria; Saccharimonadia	Saccharimonadales < 80%	0	0	0	27	0
Patescibacteria; Saccharimonadia	Saccharimonadales < 80%	20	0	13	0	0
Patescibacteria; Saccharimonadia	Saccharimonadaceae > 90%	17	0	34	0	0
Proteobacteria; Alphaproteobacteria	Micropepsaceae > 90%	0	0	0	22	0
Proteobacteria; Alphaproteobacteria	*Sphingobium czechense/rhizovicinum* 97.98%	0	0	0	30	0
Proteobacteria; Deltaproteobacteria	Polyangiaceae > 90%	0	12	0	0	0
Proteobacteria; Gammaproteobacteria	Comamonadaceae > 95%	0	0	0	39	0
Proteobacteria; Gammaproteobacteria	*Paraburkholderia mimosarum/oxyphila* 98.24%	0	0	0	13	0
Proteobacteria; Gammaproteobacteria	*Comamonas testosteroni* 99.24%	0	14	0	0	0
Proteobacteria; Gammaproteobacteria	*Citrobacter koseri* 99.49%	1444	1779	5559	72	122
Proteobacteria; Gammaproteobacteria	*Pseudomonas entomophila* 98.73%	49	151	0	45	130
Proteobacteria; Gammaproteobacteria	Sinobacteraceae > 90%	0	0	0	12	0
Proteobacteria; Gammaproteobacteria	*Frateuria* 92.95%	0	26	0	294	0
Spirochaetes; Spirochaetia	*Treponema zuelzerae* 97.21%	0	26	0	0	0
Synergistetes; Synergistia	*Thermovirga* 92.68%	28	0	24	0	0

Each line is a separate operational taxonomic unit (OTU) based on QIIME2 [23] analysis of the 16S metagenome data for the insect tissue and wood pulp in which they lived. The first two are archaea, the rest bacteria. The closest identifiable taxon to the OTU identified with BLASTn and the percentage sequence identity are given. The numbers are the number of reads from each metagenome for that OTU. If the OTU was also found in the deionized water (diH2O) negative control, the number of reads is given. OTUs found only in the control were omitted to save space

Firmicutes (Clostridia and Bacilli) formed the majority of OTUs, but most microbe species were uncommon (Table 1). Only three OTUs were found in all four experimental samples (wood pulp, midgut, hindgut, and fat body), while 30 were only found in one of the four. Two microbes dominated the *Oryctes* microbiome. More than 60% of the total OTUs were *Citrobacter koseri* (Orhi2), found predominantly in the midgut where it was 95.5% of all midgut-specific OTUs, compared to 64.4% of the hindgut OTUs and 29.5% of the fat body OTUs, and it was barely present in the wood substrate. It was also among the negative control microbes, so we cannot rule out that it is a contaminant. More than 20% of the total gut OTUs were identified as 95% similar to *Lactococcus taiwanensis* (Firmicutes: Lactobacillales)*,* though other species in the genus *Lactococcus* were similarly likely. Nearly all of these OTUs were in the fat body only, where it comprised 56.2% of the fat body OTUs. The third most common OTU in total only comprised 2.4% of total OTUs, and was Orhi1, *Bacillus cereus,* comprising 5.3% of the fat body OTUs and approximately 1% of the OTUs in the other samples. The second most common microbe in the hindgut at 9.5% of OTUs was identified as *Lysinibacillus sphaericus* (Firmicutes: Bacillales), a known entomopathogen [24], followed by *Pseudomonas entomophila* (Gammaproteobacteria: Pseudomonadales), another entomopathogen [25], at 5.5%. The latter was present in the negative control.

Two Archaea were found in the hindgut only. One is similar to *Methanobacterium beijingense* (Methanobacteria)*,* a methanogen first described in an anaerobic digester [26] and from a genus known to be digestive endosymbionts for termites [27]. The other is a new genus in Ca. Methanomethylophilaceae [28].

### Transcriptome

Paired-end RNA-Sequencing was performed on RNA extracted from the fat bodies, gastric cecae, midguts, and hindguts of four *O. rhinoceros* larvae: two males and two females. Approximately 108 million reads (or 54 million paired-end reads), or 24–30 million reads per sample, passed quality filtering totaling over 15.5 Gbp of sequences with an average read length of 143.9 (Additional file 1: Table S1). Trimming removed adapter sequences and 7289 reads with Q < 20. Overall sequencing quality of the clean data was high (Phred scores > 30) and mean base pair N content was 0.425% [29]. The coverage is more than sufficient for successful transcriptome assembly [30]. A total of 86,698 contigs (N50 = 954 bp) were assembled de novo from these reads without use of a reference genome, as none exists for this species, using CLC Genomics v7.51 (CLC Bio), which is among the leading transcriptome assemblers [30, 31]. Total percent GC of the final transcriptome covering 59.57 million bp was 38.36%, mean contig length was 687 bp, and median contig length was 402 bp. After comparing the expression in terms of read counts of all contigs between all pairs of tissues, we identified 1222 contigs differentially expressed in certain tissues relative to others (mean *p* < 0.1 for the relevant tissue pairs) (Table 2) (Additional file 2: Figure S1). This low number is expected, as the gastric cecae are projections of the midgut tissue. The hindgut and fat body showed the most significantly differentially expressed transcripts (Additional file 2: Figure S1). Blast2GO [33] successfully annotated 20,182 contigs, so manual annotation with BLAST [34] of highly and/or differentially expressed transcripts and targeted mining of the transcriptome for genes of interest supplemented the annotation (Additional file 4: Data S1).

**Table 2:** Differentially Expressed Contigs

Tissue	Over *p* < 0.05	Over 0.05 < *p* < 0.1	Under. *p* < 0.05	Under 0.05 < *p* < 0.1
Fat Body	36	175	0	37
Gastric Cecae	0	17	0	7
Hindgut	108	644	2	18
Midgut	9	38	0	0
Midgut+Cecae	0	125	0	6

Number of differentially over- or under-expressed contigs from the transcriptome per tissue type, based on the mean p-value for the comparison of the tissue or tissue pair’s expression level of a contig compared to all other tissues. Contigs under-expressed in the one tissue could alternatively be said to be over-expressed in every other tissue (ex: under-expression in the fat body means over-expression in the digestive tissue). Several contigs showed differential expression in the midgut and cecae relative to the fat body and hindgut but not compared between the midgut and cecae, which was expected as the two tissues are connected and made of the same cells developmentally [32]

**Fig. 2:**
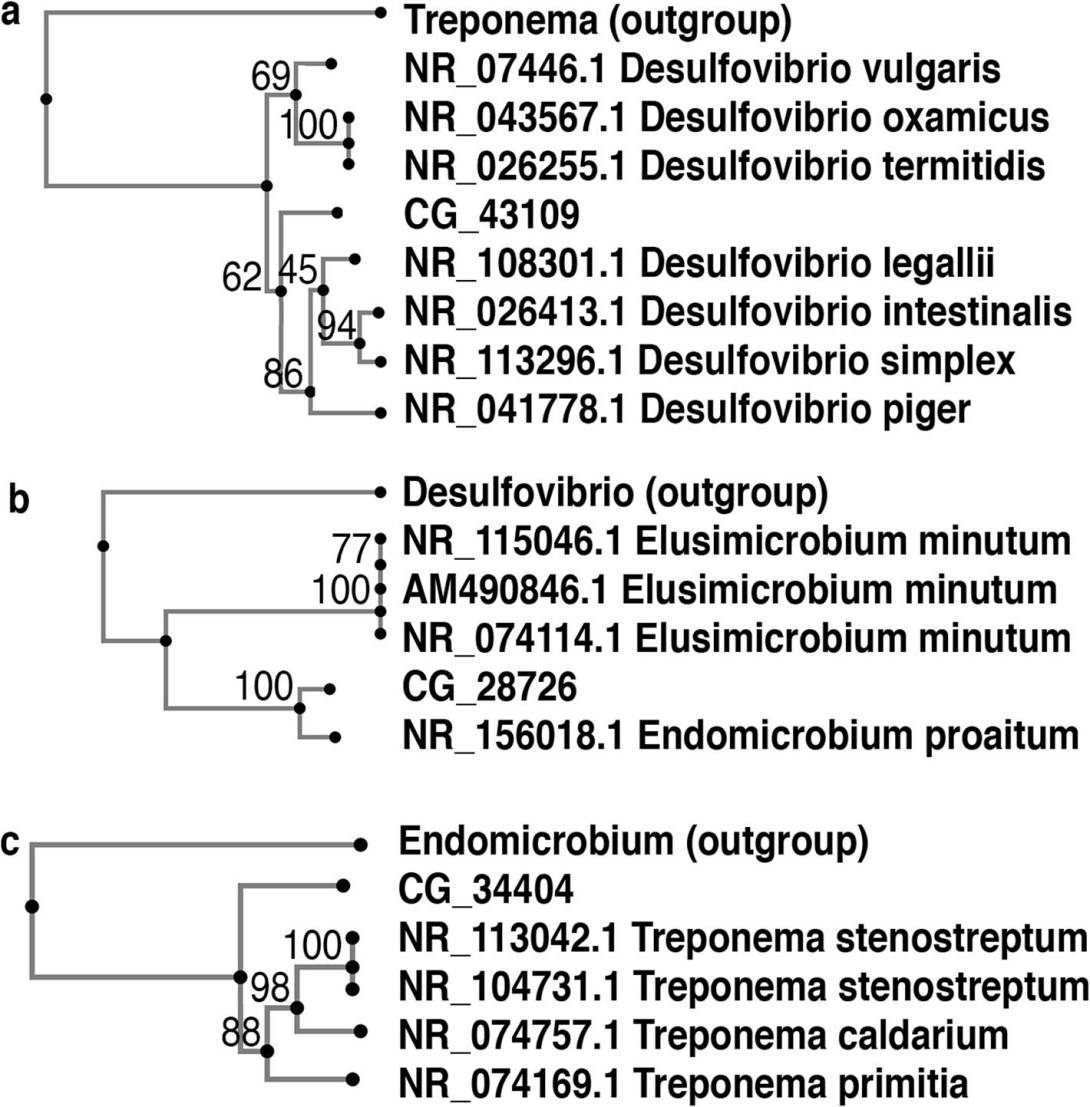
Phylogenetic Trees of Microbes Identified from the *Oryctes rhinoceros* Transcriptome. Neighbor-joining trees of the 16S ribosomal RNA sequences were generated by MAFFT v7 and rendered with Phylo.io. The GenBank *Oryctes rhinoceros* transcripts start with “CG” and the rest are the closest BLASTn hits to the transcripts, given with their GenBank Accession numbers. A) *Desulfovibrio* tree including transcript CG_43109. B) *Elusimicrobium* and *Endomicrobium* tree including transcript CG_28726. C) *Treponema* tree including transcript CG_34404

We found several transcripts belonging to microbial genes among the differentially expressed genes. These were mostly 16S ribosomal RNA, all from the hindgut, but we also found a trehalose phosphorylase [glycoside hydrolase family 65, GH65] transcript whose sequence suggested a *Mucilaginibacter* sp. origin (Bacteroidetes: Sphingobacteriales). The majority of microbial transcripts in the hindgut came from Clostridiales (Firmicutes), though we could not identify the species beyond the order. Also common were bacteria in the order Bacteroidales (Bacteroidetes). We identified several transcripts identified as *Desulfovibrio* (Deltaproteobacteria, Desulfovibrionales), a known associate of the termite gut and occasional endosymbiont of termite symbiotic protozoans [35, 36]; *Treponema* sp. (Spirochaetes, Spirochaetales), a known termite gut symbiont [37, 38]; and *Endomicrobium proavitum* (Elusimicrobia), a nitrogen-fixing microbe from a class of free-living and intracellar symbionts of termite gut protozoa [39, 40]. All are likely new species within their genera based on the < 96% sequence similarity for their 16S genes (GenBank Accession Numbers MN088856–59) to those of known species [41, 42] (Fig. 2). We also identified a ribosomal RNA transcript for a known insect gastrointestinal tract parasite, *Blastocystis* sp. (Heterokonta, Blastocystida) [43], and a uracil phosphoribosyltransferase gene from the known insect parasite genus *Gregarina* (Apicomplexa, Eugregarinorida) [44].

Some of the most highly expressed transcripts were not differentially expressed, as they were highly expressed in all or most tissues. Unsurprisingly the most highly-expressed transcript was the mitochondrial cytochrome oxidase transcript for the beetle itself. Others included ribosomal subunits, elongation factors, and several cytochrome P450s. The most highly and differentially expressed genes in the fat body were collagen, lipid-related genes like apolipophorins and fatty acyl-CoA reductase, and hexamerins (storage proteins). Several antimicrobial peptides were highly and differentially expressed in the fat body. The most highly and differentially expressed genes in the midgut were proteases (trypsin, serine protease), chitinases, lipase, and peritrophin. Many genes in the gastric cecae were similarly differentially and/or highly expressed in the midgut, and include cathepsins and tetraspanins. Most highly and/or differentially expressed genes in the hindgut were unidentifiable, but others included actin, several xenobiotic resistance genes, and all the aforementioned bacterial 16S rRNA sequences.

**Fig. 3:**
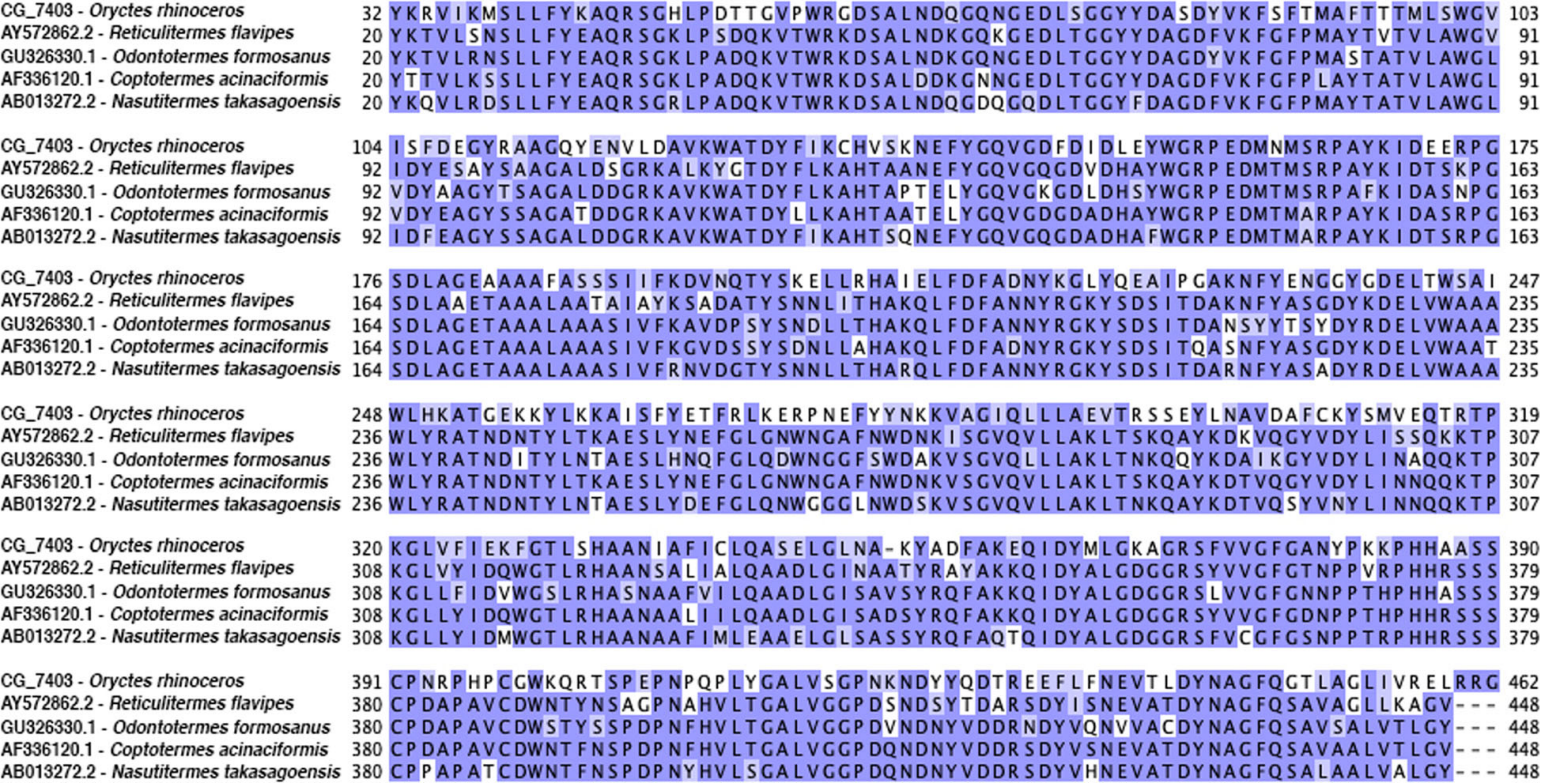
Amino Acid Sequence Similarity of the *Oryctes rhinoceros* Cellulase to Termite Cellulases. Amino acids are shaded darker with increased sequence similarity. The *Oryctes rhinoceros* cellulase (transcript CG_7403) is clearly an endogenous insect cellulase, not microbial

**Fig. 4:**
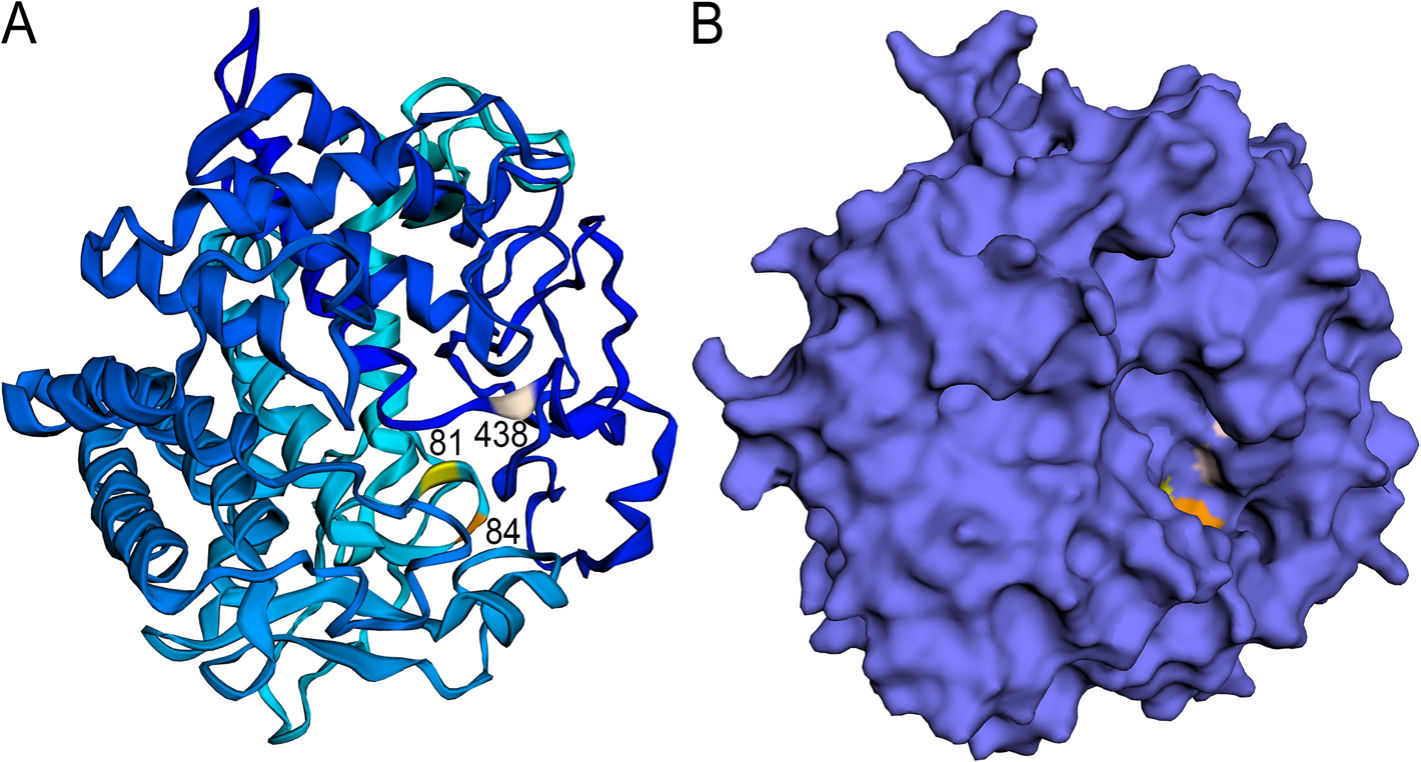
Predicted Structure of the *Oryctes rhinoceros* Cellulase. Secondary structure modeled by Phyre2 [45] with 93% of residues modeled at > 90% confidence and rendered with EzMol [46]. A) Cartoon-style backbone colored from light to dark blue from N to C terminus with the predicted catalytic site residues 81 (Aspartic Acid), 84 (Aspartic Acid) and 438 (Glutamic Acid) labeled and colored yellow, orange, and pink respectively. B) Predicted surface rendering of the protein from the same angle, with the catalytic residues colored as before

One endogenous cellulase gene (transcript CG_7403, GenBank Accession Number MN047310), with significant homology to other insect endogenous cellulases (Fig. 3), was identified in the transcriptome, but was not differentially expressed among any one tissue. Phyre2 [45] modeled 93% of the protein at 100% confidence, predicting its structure as an endo-1,4-beta-glucanase with an alpha/alpha toroid fold with six-hairpin glycosidases and a highly conserved cellulase catalytic domain (Fig. 4a). The first 30 and last 12 residues were poorly modeled, though this includes the area prior to the signal peptide. Active sites were predicted at amino acid 81 (D, Aspartic Acid), 84 (D, Aspartic Acid), and 438 (E, Glutamic Acid), using an information-theoretic approach based on Jensen-Shannon divergence [47]. These sites are located within a cleft in the protein’s predicted surface (Fig. 4b). We found no pectinases, xylanases, xyloglucanases, or lytic polysaccharide monooxygenases. We found multiple glycoside hydrolase (GH) family 1 transcripts with close amino acid sequence similarity to insect cellobiase [beta-glucosidase] or lactase-phlorizin hydrolases compared to insect myrosinase or microbial GH1s (Additional file 3: Figure S2).

We found several antimicrobial peptide genes. Differentially and highly expressed in the fat body were oryctin, rhinocerosin, and two attacin transcripts, with another attacin more common in the fat body but not significantly, plus two defensins with low expression (Table 3). Differentially and highly expressed in the midgut was thaumatin. We also uncovered a large amount of transcripts for the defense and xenobiotic resistance proteins cytochrome P450, glutathione-S-transferase, and carboxylesterase; as well as peptidoglycan-recognition and toll-pathway proteins involved in immune cascades. Some were differentially and/or highly expressed in certain tissues, particularly the fat body, but the majority was spread throughout these tissues (Additional file 4: Data S1). The tissue with the least expression of these genes was the hindgut.

**Table 3:** Antimicrobial Peptides of *Oryctes rhinoceros*

Contig ID	Annotation	Raw Expression Values (Reads /1 K Base Pair)				GenBank Accession
		Fat Body	Midgut	Hindgut	Gastric Cecae	
CG_21477	Attacin•	3624	42	37	77	MN047305
CG_29216	Attacin•	608	18	7	16	MN047306
CG_32953	Attacin	715	58	91	19	MN047304
CG_42418	Defensin	4	4	15	9	MN047302
CG_55756	Defensin	0	0	53	0	MN047303
CG_81916	Defensin	23	0	0	0	MN047301
CG_17671	Oryctin•	51,453	126	2267	194	MN047308
CG_17845	Rhinocerosin•	5114	52	160	62	MN047309
CG_2230	Thaumatin•	35	59,375	42	1960	MN047307

Significant differential expression noted as follows: *p* < 0.1 = •, *p* < 0.05 = *

## Discussion

Certain microbes do seem to be more prevalent in the *Oryctes* body compared to the environment. Both *Bacillus cereus* and *Citrobacterer koseri* were found in the one previous, culturing-based study of the *Oryctes* rhinoceros gut by Sari et al [19], and a *Citrobacter* and *Bacillus* were also isolated in a recent study using cellulase-agar to selectively enrich cellulolytic microbes [20]. The possibility exists that *Citrobacter koseri* is a contaminant in our samples, however, as it was present in the negative control. *Citrobacter* species are notoriously cosmopolitan, so we cannot conclude whether or not our samples or even those of past researchers were contaminated, or whether *Citrobacter koseri* is a genuine *Oryctes* gut resident. The point is likely moot, as its ubiquity would mean it is not an essential symbiont but a transient gut microbe. Alternatively, the species is not *Citrobacter koseri*, but a conserved *Oryctes rhinoceros* symbiont in the same genus that cannot be differentiated from *Citrobacter koseri* on the basis of 16S gene sequence alone. Fatty acid methyl ester analysis would rule this out. The *Pseudomonas entomophila* OTU in our sample meanwhile is likely a contaminant, despite that species being a known insect gut inhabitant as its name suggests [25]. No other OTU from the gut or wood samples was found in the negative control, so we are confident in their natural associations with the insect.

The molecular data identified microbes associated with termite guts, including archaea as well as bacteria. Some may be intracellular symbionts of flagellate gut symbionts or other protozoa. Some have known or putative celluolytic abilities or interact with cellulolytic microbes, such as *Treponema* [48], *Bacillus cereus*, and *Citrobacter koseri* [19]. Undoubtedly many of these species assist in digestion, as in termites, though the beetles may not necessarily depend on them for survival. A member of the recently described phylum Elusimicrobia lives in the *Oryctes rhinoceros* gut as well, either free-living or as an ecto- or endo-symbiont within another, protozoan symbiont. The first cultivated member of the phylum*, Elusimicrobium minutum*, was isolated from a related humivorous scarab beetle, *Pachnoda ephippiata* [49], however the *Oryctes* sequence is closer to the nitrogen fixing *Endomicrobium proavitum* found in termite guts [39, 40]. The *Oryctes* Elusimicrobia 16S ribosomal RNA transcript (CG_28726) is 96.55% similar to that of *Endomicrobium proavitum* Strain Rsa215 (GenBank Accession Number NR_156018.1), and may be a new species of *Endomicrobium*, though given the short length of the amplicon one cannot be certain of that at this time. The possibility that it can be cultured under the right conditions recommends future efforts to do just that. *Oryctes rhinoceros* likely also houses a potentially new species of *Treponema,* found in the metagenomics and transcriptomics datasets alike. We hypothesize based on the transcriptome data that a species of *Blastocystis* is the dominant protozoan symbiont of the *Oryctes* gut, but cannot currently attribute any digestive functions to it, nor are we proposing any obligate symbioses with conserved vertical or horizontal transfer of the protozoan. FISH probes for these species will be designed and used to understand their ecology better, as a necessary prerequisite to assigning a *Candidatus* binomial name to them [50]. Termite gut microbiomes tend to be consistent within the species [51], so it would be interesting to see how the microbiomes of *Oryctes rhinoceros* compare across their range in the Pacific.

The uncultured *Lactococcus* species, absent in the negative control and found predominantly in the fat body according to the metagenomics data, is the most likely candidate for an endocellular symbiont, but there is no precedence for such symbiosis in *Lactococcus*. Species of *Lactococcus* have, however, been isolated from the guts of wood-feeding termites [52]. We did not find evidence for it or *Citrobacter koseri* in the fat body transcriptome. This would be expected if the microbes are extracellular or otherwise would have been washed out of the tissues prior to RNA extraction. This raises the possibility that the two are not fat body microbes at all, but hemolymph microbes [53] and/or contaminants from injury to the gut during dissection despite our efforts to prevent this. Unfortunately few to no papers studying arthropod hemolymph microbiota have been published for us to check for precedent.

We found several species of Clostridiales bacteria in both the transcriptome and the microbiome data, though we could not accurate identify them to family in most cases. Whether the metagenome and transcriptome sequences refer to the same microbes or not is likely but cannot be determined with absolute certainty: ultimately the results of such molecular biology assays depends both on the software used to assemble the genome/transcriptome libraries as well as the availability of related genes in the respective databases [54]. The presence of Clostridiales microbes in the hindgut of a wood-feeding insect is itself not surprising, as the class includes several anaerobes and organic matter fermenters and has been reported in termite guts [55]. Along with the *Treponema*, also known from termites [56], the Clostridiales microbes may assist in digestion of the otherwise recalcitrant wood pulp. Harder to explain is the abundance of Clostridiales 16S genes in the fat body metagenome, when the transcriptome data suggests they would be limited to the hindgut, unless you assume the fat body sample was contaminated with hemolymph microbes as mentioned earlier. The species *Clostridium bifermentans* is a known pathogen of mosquitoes, so entomopathogenic Clostridiales in the hemolymph have precedent [57]. The fat body metagenome data may thus be unreliable due to hemolynph contamination. Future extractions should use intensive washing to remove the hemolymph and pair them with culturing and metagenomic analysis of the hemolymph itself and/or in situ hybridization tests to visualize in which tissues these specific microbes are located [58]. In addition, future work should look at the adult beetle microbiome, to see if and how the gut microbiota changes after metamorphosis and to develop hypotheses for possible vertical transmission of certain symbionts.

We only found one true cellulase in the transcriptome: a GH9 beta-1,4-endoglucanase (transcript CG_7403) with sequence homology to other endogenous insect cellulases (Fig. 3) [59]. The enzyme has a highly conserved cellulolytic catalytic domain (Fig. 4a) located within a cleft (Fig. 4b), as is typical of endoglucanase cellulases [60]. We thus have strong reason to believe that *Oryctes rhinoceros* produces its own cellulase, and can at least partially break down cellulose without microbial symbionts. However, this enzyme transcript was not highly or differentially expressed in any tissues. If the beetles had not been feeding prior to RNA extraction, this would be expected. Previous research with a related species, *Oryctes nasicornis*, detected cellulase activity but failed to produce “soluble cellulase” and concluded the cellulolytic ability of that species was microbial in origin [61, 62]. Previous work with *Oryctes rhinoceros* isolated a cellulolytic *Citrobacter* and lignolytic *Bacillus* microbe [20]. Cellulolytic microbes have also been found in other Scarabaeidae beetles, of which the only one related to the microbes we found was *Citrobacter freundii* [63]. Endogenous insect cellulases and microbial cellulases are not mutually exclusive, so there is no reason to assume an organism must only have one or the other. At this point in time the evidence points to both an expressed endoglucanase enzyme gene in the *Oryctes rhinoceros* genome and a microbiome with cellulolytic bacteria, most likely species of *Citrobacter*.

Complete cellulose digestion also requires cellobiase or beta-glucosidase, which is in the GH family 1 [62]. We identified several endogenous insect GH1 sequences in the transcriptome, but it is unclear how many are true cellobiases and how many are other GH1s like myrosinase, galactosidase, or lactase/phlorizin hydrolase (Additional file 3: Figure S2). We did identify a putative bacterial cellobiase (transcript CG_62808), but it was truncated and had < 50% sequence identity to any known sequences in the NCBI database. We hypothesize that the beetle’s own cellulase enzyme works synergistically with endogenous and/or microbial beta-glucosidases to fully break down wood to glucose, as occurs in termites [64]. Future research will need to untangle what digestive enzymes are active in the hindgut of these beetles, and whether the sources are endogenous to the insect or microbial [14]. Observing how gene expression changes in the adult is also an important next step.

The high and/or differentially expressed genes in the different tissues (Additional file 4: Data S1) matched what we know about these organs’ functions: fat transport and storage in the fat body, peritrophic membrane production and digestion in the midgut, etc. Protein digestion seems to occur primarily in the midgut, but we cannot conclude where carbohydrate breakdown dominates: perhaps our larvae had not been eating prior to RNA extraction, despite having full guts and being surrounded by edible substrate. That would also explain the low expression of cellulase.

The xenobiotic defense genes are scattered throughout the transcriptome: some are tissue specific, others expressed in multiple tissues, though less likely in the hindgut. Antimicrobial peptide production is highest in the fat body and/or hemolymph, as is typical for insects [65]. Unsurprisingly we found high expression of the eponymous peptides oryctin and rhinocerosin, first discovered in *Oryctes rhinoceros* [9, 10]. The only antimicrobial peptide not produced in the fat body was thaumatin, which was highly and differentially expressed in the midgut (Table 3). Thaumatin is an antifungal peptide related to insect defensins that is known in the model beetle *Tribolium castaneum* but absent from other insects like *Drosophila*, *Anopheles*, and *Apis* [66]. The expression of a peptide that kills filamentous fungi in the midgut could help prevent mold from growing in the food before it is fully digested in the hindgut, or could be an evolved defense against entomopathogenic fungi (Maistrou, 2018 #175). Reduced antimicrobial peptide production in the hindgut could have evolved to reduce harm to the symbionts, as is the case in aphids [67]. This finding combined with the abundance of microbe transcripts in the hindgut leads us to suspect *Oryctes rhinoceros* has an at least facultative relationship with its hindgut flora. As the insects we cultured had not been given an immune challenge, their expression of immunity genes is not expected to be highly elevated and there may be more antimicrobial genes in the *Oryctes rhinoceros* repertoire that our analyses would have missed.

## Conclusion

The culturing, DNA metagenomics and RNA transcriptomic data combine to give us meaningful yet at times differing conclusions about the microbial community of *Oryctes rhinoceros.* These are known complications of the various methods of studying microbiomes [54], so repeated analyses of the *Oryctes rhinoceros* microbiome are needed to see which findings hold. The evidence points towards symbioses similar to those of the termite gut, and to several potentially new species to be determined with anaerobic culturing and microscopy. Chemical or proteomic tests of the gut enzymes and genomic tests for the presence of PCWDE genes will help identify how the larvae break down their recalcitrant wood diet. We have identified several genes involved in microbial, chemical, and xenobiotic resistance that we add to the knowledge of this pest in the quest to develop suitable controls, and to the growing database of antimicrobial peptides. Lastly, our publically deposited transcriptome assembly data greatly increases the amount of molecular data available for this agriculturally important organism. Our data is a foundation for future research, both basic studies on *Oryctes* biology and potential RNAi studies geared towards pest control.

## Methods

### Insects and dissection

Wild, larval *Oryctes rhinoceros* were collected from decaying coconut (*Cocos nucifera* L.) logs in public land in Jiuru Township, Pingtung County, Taiwan (22.722600°N, 120.510506°E). No permissions or consent were required to use this pest species in our study. Two adults were collected and are kept as voucher specimens at the Department of Entomology at National Taiwan University. The fat bodies and digestive tracts were dissected from four late-instar larvae (two male, two female) that had been feeding on coconut log pulp until dissection. The midgut and hindgut contents were removed and 30 mg of each mixed with phosphate-buffered saline (PBS). Samples of fat body and the wood pulp substrate the larvae lived in were also mixed with PBS on ice. Samples of fat body and the washed gut tissues from the four larvae divided into gastric cecae, midguts, and hindguts were stored as one pool per tissue in 10x volume of RNA Later at − 80 °C overnight until RNA extraction could be performed. Four pooled larvae is more than sufficient for this type of experiment, as prior beetle larval transcriptomes were performed with as few as one larva [68].

### Microbiology

From the samples in PBS, 50 μL was used immediately to inoculate petri dishes of nutrient agar (HiMedia® Laboratories Pvt. Ltd.) under a laminar flow biosafety hood, and the rest used for DNA extraction and microbiome analysis. Petri dishes were incubated at 30 °C. Isolated pure colonies of cultured microbes were lysed in 50 μL DNase/RNase-free water for 10 min at 95 °C and PCR performed for the 16S rDNA region with the protocol and primers [27F, AGAGTTTGATCMTGGCTCAG, and 1492R, CGGTTACCTTGTTACGACTT] as described in Shelomi, 2019 [69]. The PCR products were sent for sequencing to Mission Biotech Co, Ltd. (Taiwan), using a Thermo Fisher Scientific BigDye® Terminator v3.1 Cycle Sequencing Kit, Applied Biosystems 3730xl DNA Analyzer, and Beckman Coulter Biomek® NX Laboratory Automation Workstation (http://www.missionbio.com.tw). The resulting forward and reverse sequences were viewed with 4Peaks v1.8 (https://nucleobytes.com/4peaks/index.html), merged with EMBOSS merger [70], and compared to known 16S rDNA sequences with BLASTn [34].

DNA was then immediately extracted from the pooled midgut contents, hindgut contents, fat body, and wood pulp using the DNeasy PowerWater Kit. These four tissue pools as well as a negative control sample of deionized water routinely used in the laboratory were sent for full 16S rRNA metagenomics at BioTools Co., Ltd. (Taiwan). The quality control, library construction, sequencing (paired-end Illumia HiSeq 2000, 250 bp paired-end reads), and resulting identification of the operational taxonomic units (OTUs) using QIIME2 [23] were all as described in Shelomi 2019 [69]. The raw data was uploaded to the NCBI Short Read Archive (Accession number SRR9208133–6).

### Transcriptomics

RNA was separately extracted from the fat bodies, hindguts, midguts, and gastric caecae of the four pooled larvae using the TRIZol protocol [71] with 1-bromo-3-chloropropane instead of chloroform. RNA quality was measured with a NanoDrop™ spectrophotometer. RNA was then sent to TechComm Next Generation Sequencing Core for RNA library construction (mRNA polyAbase) and sequencing (Illumina HiSeq 4000, paired end 150 bp). Adapter sequence and quality control (>Q20, error rate < 1%) trimming was done with Trimmomatic [72]. Quality control was done with FastQC and compiled with MultiQC v1.5dev0 [29] (Additional file 1: Table S1). The transcriptome was de novo assembled and the expression levels of each contig in the different tissues calculated using CLC Genomics v7.51 (CLC Bio). The parameters used were as follows: Mapping mode = Map reads balls to contains (slow); Update contains = Yes; Automatic bubble size = Yes; Minimum contain length = NA; Automatic word size = Yes; Perform scaffolding = Yes; Auto-detect paired distances = Yes; Mismatch cost = 2; Insertion cost = 3; Deletion cost = 3; Length fraction = 0.5; Similarity fraction = 0.8; Create list of un-mapped reads = no. Open reading frame prediction was performed by ContigViews system [73]. The raw data was uploaded to the NCBI Short Read Archive (Accession number SRR9208137–40) and the assembled transcriptome to the NCBI Transcriptome Shotgun Assembly Sequence Database (Accession number GHNO01000000).

The significance tests were performed in R (version 3.5.1). The size factors were first calculated to normalized read counts for all samples. We defined as “differentially expressed” any transcript whose mean *p*-value for significant difference in expression levels according to the likelihood ratio test [74] was < 0.1 in all pairs involving that tissue. For example, if the comparison of expression levels of a transcript had *p*-values < 0.1 for fat body to gastric cecae, fat body to hindgut, and fat body to midgut, then the transcript is differentially expressed in the fat body. The size-factor calculation and likelihood ratio test were both performed using DESeq function of DESeq2 package [75] in R by setting “test = ‘LRT’” and “reduced = ~1” while using the default values for the other parameters. A heatmap for the normalized read counts for only the differentially expressed contigs was made using Heatmapper [76] with complete linkage clustering with the Pearson distance measurement method applied to the columns (Additional file 3: Figure S2).

We annotated the transcriptome using Blast2GO’s [33] built-in tblastx program to compare each sequence to the NCBI translated nucleotide database, with an expect value threshold of e^− 6^. Contigs with successful BLAST [34] hits were mapped to the Gene Ontology (GO) database and annotated using Blast2GO with an expect value threshold of e^− 6^. In addition, all differentially expressed transcripts and the top 100 most highly expressed transcripts per tissue type were manually BLAST-ed [77] to the non-redundant (nr) protein database and the 16S ribosomal DNA sequence [78] to identify the transcripts as accurately as possible (accessed 31 May 2019). The transcriptome was mined with tBLASTx for antimicrobial peptides, cytochrome P450’s, glutathione-S-transferase, carboxylesterases, and other xenobiotic resistance and detoxification genes to understand their defenses against microbial pathogens, plant semichemicals, and pesticides [16]; and plant cell wall degrading enzymes such as cellulases, hemicellulases, pectinases, and lytic polysaccharide monooxygenases to understand their ability to digest plants, using query statements of relevant insect genes downloaded from the NCBI nucleotide database [77] as has been done in other studies [13, 79]. Hits were translated to amino acid sequences with ExPASy [80], their identity confirmed with a BLASTp search of the NCBI database, and signal peptides identified with SignalP 5.0 [81]. The sequence data was aligned with MAFFT v7 [82, 83] using the G-INS-i iterative refinement method [84], and BLOSUM62 scoring matrix while leaving gappy regions. An average linkage UPGMA guide tree was calculated with the MAFFT online system [83] using the WAG substitution model ignoring heterogeneity among sites. Because many transcripts were truncated, fragmentary sequences were clipped with MaxAlign [83, 85] then the remaining gap-free sites used to make a neighbor joining tree with the WAG substitution model, ignoring heterogeneity among sites, and with bootstrapping over 1000 trees. Trees were viewed with Phylo.io version 1.0.0 [86].

For any found cellulase, we searched the structural database of the Phyre2 Protein Fold Recognition server (http://www.sbg.bio.ic.ac.uk/phyre2/) to form a predictive model of its structure [45], and used the EzMol interface [46] to render the protein structure (Fig. 4). We aligned the cellulase with other endogenous insect cellulase genes using MUSCLE [87] and JalView [88]. For any microbial 16S ribosomal RNA genes identified from the transcriptome that we were able to identify past family level, we compared their nucleotide sequences to those of closely related species plus one outgroup with MAFFT v7 using the G-INS-i iterative refinement method as above, clipped fragmentary sequences with MaxAlign, and calculated a neighbor joining tree of the conserved sites with the Jukes-Cantor model and bootstrapping over 1000 trees (Fig. 2). Any non-truncated GH1s were aligned with insect and microbial GH1s (beta-glucosidases or cellobiases, myrosinases, and lactase-phlorizin hydrolases) with MUSCLE and JalView, then a phylogenetic tree made with MAFFT v7 as above (Additional file 3: Figure S2).

## Supplementary information

**Additional file 1: Table S1.** General fastq and FastQC statistics for the *Oryctes rhinoceros* RNA-Seq. Data produced by MultiQC v1.5.dev0 [29].

**Additional file 2: Figure S1.** Heatmap of differentially expressed contigs in *Oryctes rhinoceros* tissues. Heatmap made with Heatmapper [76] for the 1222 differentially expressed contigs only, based on normalized read counts (Additional file 4: Data S1). Rows represent contigs ordered according to complete linkage clustering with the Pearson distance measurement method applied to the columns representing the four tissue types. Red areas are underexpressed while blue areas are overexpressed. The figure shows distinct expression patterns for the four tissues, with greater similarity between the midgut and gastric cecae. FB=Fat Body. GC = Gastric Cecae. HG = Hindgut. MG = Midgut.

**Additional file 3: Figure S2.** Phylogeny of *Oryctes rhinoceros* Glycoside Hydrolase 1 Transcripts. Neighbor-joining trees of the GH1 ribosomal RNA sequences were generated by MAFFT v7 and rendered with Phylo.io. The *Oryctes rhinoceros* GH1s start with “CG.” Only those with complete open reading frames were used. Note that CG_365 was a single transcript coding for what appeared to be two separate GH1 genes between one start and stop codon.

**Additional file 4: Data S1.** Annotations and differential expression statistics for *Oryctes rhinoceros* tissues. Putative annotations based on Blast2GO of the full transcriptome including UniProt ID, and manual BLAST results of highly or differentially expressed transcripts. All non-insect transcripts are noted in bracketed descriptions. “#N/A” means Blast2GO failed to annotate the transcript. “Unidentifiable” means there were no hits, the results were overly ambiguous, or the hits were only to unidentified hypothetical proteins even after manual BLAST to the NCBI database. Expression values are given in reads per kilobase per million mapped reads. Contigs are differentially expressed in a tissue or pair of tissues if the mean *p*-value for the difference between the normalized expression values of for all pairs with that tissue or tissue pair is less than 0.1, and significantly differentially expressed (marked with an*) if the mean *p* value is < 0.05. The next column notes if this significance refers to over- or under-expression relative to others. Contigs are marked as whether or not they are highly expressed if their raw expression value in the tissue where there are differentially expressed [or mean expression value for pairs] is in the top 1% (•) or 0.01% (••). FB=Fat Body. GC = Gastric Cecae. HG = Hindgut. MG = Midgut.

## Data Availability

The transcriptome and 16S metagenomics raw data has been uploaded to the NCBI Short Reads Archive, Accession Numbers SRR9208133–40. This Transcriptome Shotgun Assembly project has been deposited at DDBJ/EMBL/GenBank under the accession GHNO00000000. The version described in this paper is the first version, GHNO01000000. Annotated nucleotide sequences are available on GenBank for the AMPs (MN047301–9), cellulase (MN047310), cultured bacteria (MN089572–4), and bacteria identified from the transcriptome (MN088856–59).

## Abbreviations

AMP: Antimicrobial Peptides
ATCC: American Type Culture Collection
bp: Base pair
FB: Fat body
FISH: Fluorescence in situ hybridization
Gbp: Giga base pairs (1Gbp = 1000000000 base pairs)
GC: Gastric cecae
GH: Glycoside hydrolase
HG: Hindgut
MG: Midgut
NCBI: National Center for Biotechnology Information
nr: Non-redundant
OTU: Operational taxonomic unit
PBS: Phosphate-buffered saline
PCWDE: Plant cell wall degrading enzyme
RNAi: RNA interference
RNA-Seq: RNA sequencing
WAG: Whelan and Goldman
