# Supplementary figures and images for: Transcriptome and microbiome of coconut rhinoceros beetle (Oryctes rhinoceros) larvae
Source: BMC Genomics. 2019 Dec 9;20:957. doi: 10.1186/s12864-019-6352-3 (PMC6902462; doi:10.1186/s12864-019-6352-3)

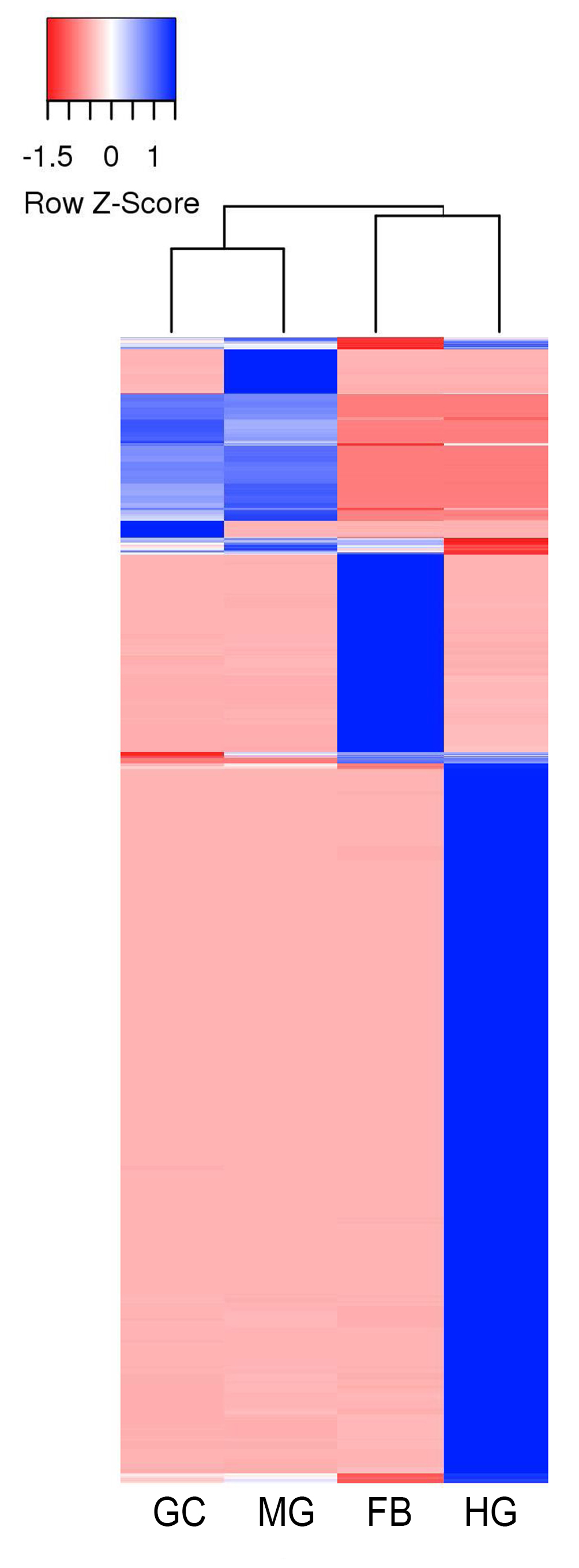

Supplement: Supplementary file 2 — Additional file 2: Figure S1. Heatmap of differentially expressed contigs in Oryctes rhinoceros tissues. Heatmap made with Heatmapper [76] for the 1222 differentially expressed contigs only, based on normalized read counts (Additional file 4: Data S1). Rows represent contigs ordered according to complete linkage clustering with the Pearson distance measurement method applied to the columns representing the four tissue types. Red areas are underexpressed while blue areas are overexpressed. The figure shows distinct expression patterns for the four tissues, with greater similarity between the midgut and gastric cecae. FB=Fat Body. GC = Gastric Cecae. HG = Hindgut. MG = Midgut. [file 12864_2019_6352_MOESM2_ESM.jpg]

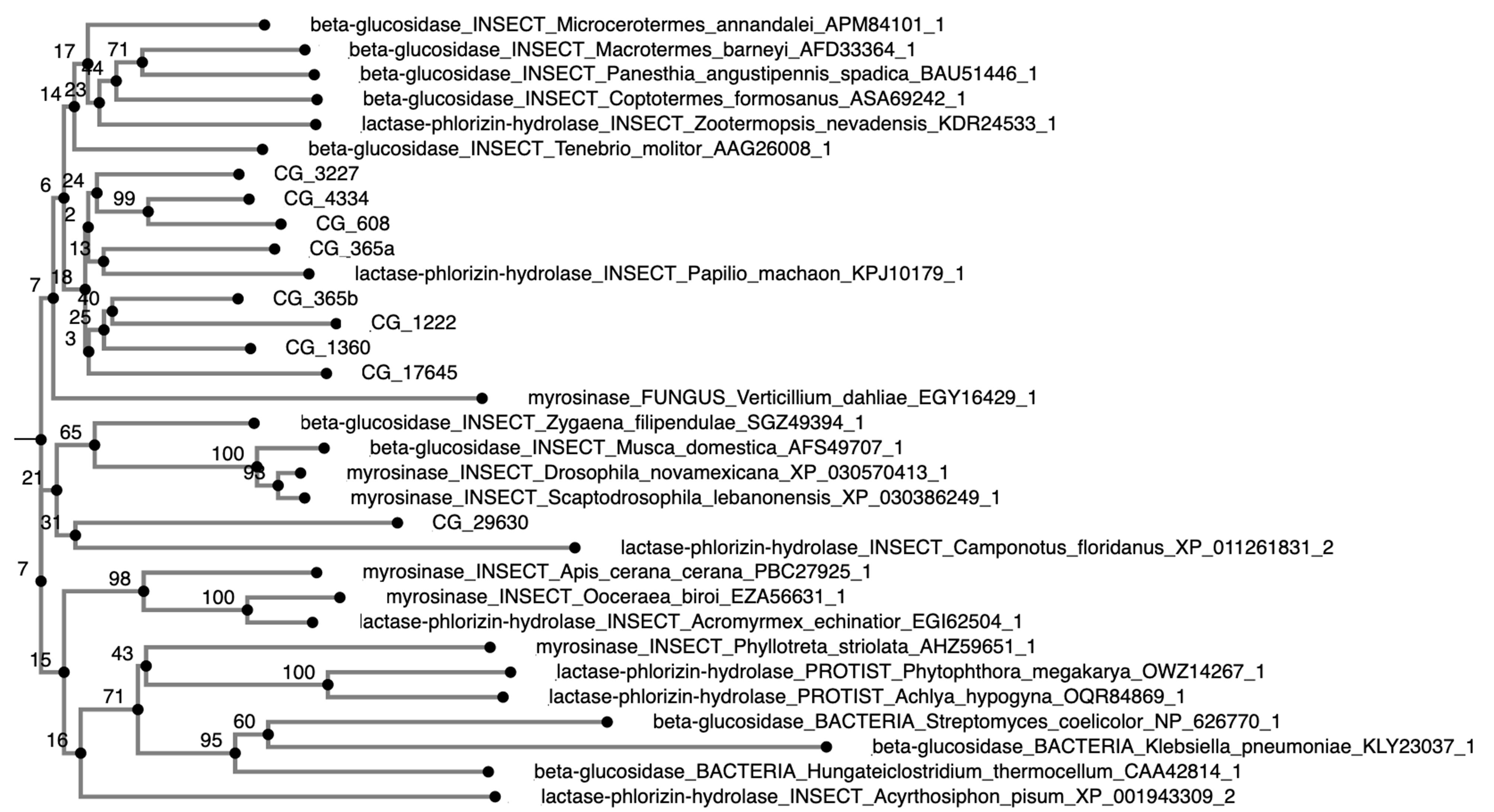

Supplement: Supplementary file 3 — Additional file 3: Figure S2. Phylogeny of Oryctes rhinoceros Glycoside Hydrolase 1 Transcripts. Neighbor-joining trees of the GH1 ribosomal RNA sequences were generated by MAFFT v7 and rendered with Phylo.io. The Oryctes rhinoceros GH1s start with “CG.” Only those with complete open reading frames were used. Note that CG_365 was a single transcript coding for what appeared to be two separate GH1 genes between one start and stop codon. [file 12864_2019_6352_MOESM3_ESM.jpg]
